# Use of antimicrobial resistance information and prescribing guidance for management of urinary tract infections: survey of general practitioners in the West Midlands

**DOI:** 10.1186/s12879-016-1559-2

**Published:** 2016-05-24

**Authors:** Dean Ironmonger, Obaghe Edeghere, Savita Gossain, Peter M. Hawkey

**Affiliations:** Field Epidemiology Service, Public Health England, 5 St Philips Place, Birmingham, UK; Public Health Laboratory, Public Health England, Heart of England NHS Foundation Trust, Birmingham, UK; Institute of Microbiology and Infection, Biosciences, University of Birmingham, Birmingham, UK

**Keywords:** Antimicrobial resistance, Antibiotic prescribing, Urinary tract infection, Community sampling

## Abstract

**Background:**

There is a marked variation in both antibiotic prescribing practice and urine sampling rates for diagnostic microbiology across general practices in England. To help understand factors driving this variation, we undertook a survey in 2012/13 to determine sampling protocols and antibiotic formularies used by general practitioners (GPs) for managing urinary tract infections (UTIs) in the West Midlands region of England.

**Method:**

Cross-sectional survey of all eligible general practices in the West Midlands region of England undertaken in November 2012. GPs were invited to complete an online survey questionnaire to gather information on policies used within the practice for urine sampling for microbiological examination, and the source of antibiotic formularies used to guide treatment of UTIs. The questionnaire also gathered information on how they would manage five hypothetical clinical scenarios encountered in the community.

**Results:**

The response rate was 11.3 % (409/3635 GPs), equivalent to a practice response rate of 26 % (248/950). Only 50 % of GPs reported having a practice policy for urine sampling. Although there was good agreement from GPs regarding collecting specimens in scenarios symbolising treatment failure (98 %), UTI in an adult male (98 %) and asymptomatic UTI in pregnancy (97 %), there was variation in GPs requesting a specimen for the scenarios involving a suspected uncomplicated urinary tract infection (UTI) and an asymptomatic catheterised elderly patient; with 40 and 38 % respectively indicating they would collect a specimen for microbiological examination.

**Conclusion:**

Standardised evidence based clinical management policies and antibiotic formularies for GPs should be readily available. This will promote the rational use of diagnostic microbiology services, improve antimicrobial stewardship and aid the interpretation of ongoing antimicrobial resistance surveillance.

**Electronic supplementary material:**

The online version of this article (doi:10.1186/s12879-016-1559-2) contains supplementary material, which is available to authorized users.

## Background

Antimicrobial resistance (AMR) is a serious threat to health and the inappropriate use of antibiotics is central to the development of antibiotic resistance [1]. The UK five year AMR Strategy recommends the strengthening of AMR surveillance to inform local prescribing and enable the monitoring of the impact of interventions aimed at reducing the burden of antibiotic resistance [2].

Antimicrobial susceptibility data from diagnostic microbiology laboratories can be used for surveillance to monitor trends in AMR [3]. This data is based on specimens submitted to laboratories and may be subject to selection bias due to over sampling of clinical specimens from patients with initial treatment failures, complicated clinical histories or severe infections [4, 5]. An English study in 2004 found a wide range for sampling urine specimens, from 29 to 266 urine samples/1000 patients/year [5]. A Welsh study in 2006 found a similar range, with sampling rates varying from 0.6 to 237.3 urine samples/1000 patient/year [4], suggesting substantial variability in local sampling policies.

Studies in England and the United States have shown urine is the most frequent specimen sent for microbiological examination from non-hospitalised patients and urinary tract infection (UTI) is one of the most common diagnoses that results in antibiotic prescribing [6, 7]. In England in 2009, there was a fivefold difference in antibiotic prescribing volume between general practices [8], with 74 % of antibiotic prescribing occurring in community settings in 2014 [9].

There is a linear relationship between trends in antibiotic consumption and resistance [10, 11]. Variation in antibiotic prescribing rates in general practices have been shown to be negatively associated with variation in observed antibiotic resistance in the local population [12, 13]. National guidance for the management of infections and prescribing in the community has not reduced the variation in antibiotic prescribing across general practices in the UK, particularly in the management of upper respiratory and urinary tract infections [14].

In order to better understand some of the organisational and behavioural factors driving this variation in both antibiotic prescribing and specimen sampling for diagnostic microbiology, and thereby aid the interpretation of routine AMR surveillance data, we conducted a survey among general practitioners (GPs) in the West Midlands to examine the use of diagnostic microbiology services and empirical treatment for patients presenting with suspected UTI, and determine the source of prescribing advice.

## Methods

### Setting/population

The West Midlands is one of nine English administrative regions and in 2012 there were 950 general practices with a total of 3635 general practitioners responsible for 5.8 million registered patients [15]. Each practice had an average of 4 GPs with an average practice list size of just over 6,000 patients and 73 % of practices were located in Local Authority (English local administrative unit) areas designated as urban [15].

### Survey of GPs in the West Midlands

We conducted a cross-sectional survey during November 2012 to February 2013 among GPs providing community healthcare in the West Midlands. Community healthcare was defined as ambulatory primary healthcare delivered by registered GPs working within practices in the West Midlands.

The survey questionnaire was developed using a template from an earlier Welsh study [4] and consisted of 17 questions divided into 3 sections (Additional file 1). Section 1 collected demographic data related to the practice and GPs. Section 2 elicited information on policies employed for the management of UTI, with questions on the use and source of prescribing formularies, existence of practice policies for urine sampling; how microbiological results influenced antibiotic prescribing; and an estimate of the proportion of patients clinically suspected as having a UTI for which urine specimens were requested.

Section 3 described five hypothetical clinical scenarios (A to E) involving potential UTI presentations and GPs were asked whether they would request a specimen and/or prescribe antibiotics empirically (Table 1). Section 4 captured free text comments from the respondents.

**Table 1:** Clinical scenarios presented to survey participants

Scenario A: Treatment failure in a young woman A 20 year old lady re-attends surgery and complains that the loin pain and frequent urination symptoms reported to you the previous week had worsened despite finishing a complete course of trimethoprim (no sample was taken previously).	
Scenario B: Probable uncomplicated UTI A 43 year old woman complains of pain passing urine and frequency. She feels well otherwise and has not previously been treated for a UTI.	
Scenario C: Probable UTI in an adult male A 51 year-old man attends your surgery complaining of pain passing urine and perineal tenderness. On examination you find suprapubic tenderness and a temperature of 38.5 C is measured.	
Scenario D: Possible asymptomatic UTI in pregnancy During a routine antenatal clinic an 18 year old girl who is 20 weeks pregnant produces a cloudy urine sample. She reports no symptoms or discomfort. The urine dipstick tests positive for nitrite but negative for leukocytes and protein.	
Scenario E: Catheterised asymptomatic elderly female You visit an 82 year old female in a nursing home. She is catheterised, afebrile and has no symptoms but the staff inform you that the urine is cloudy.	

We obtained a sampling frame of all eligible practices and GPs from NHS Primary Care Trusts (PCTs) (healthcare commissioners, now replaced by Clinical Commissioning Groups). This sampling frame contained email addresses of GP Practice Managers within the localities of 10 of the 17 PCTs in existence at the time of the study, with the remaining PCTs agreeing to use their existing networks to disseminate the survey invitation.

In October 2012, five GP practices were randomly selected from the sampling frame and invited to pilot the questionnaire. Two of these practices, consisting of 20 registered GPs participated in the pilot and the feedback received was used to improve the questionnaire.

The final questionnaire was produced and hosted online using SelectSurvey.net (ClassApps, USA). No sample size calculation was undertaken as all eligible practices were invited to complete the survey via email during November 2012. One email reminder was sent out to practices in January 2013 and the survey closed in February 2013.

### Statistical analysis

The survey data was collated using Microsoft Excel (Microsoft Redmond, WA). Categorical variables were summarised as counts and proportions with differences between male and female GPs tested using a two-proportion Z test with *p* < 0.05 considered statistically significant. All statistical analyses were performed using STATA v12 (StataCorp, USA). All free text comments were analysed by thematic analysis.

## Results

The response rate was 11.3 % (409/3635 GPs) equivalent to a practice response rate of 26 % (248/950). The age group distribution of respondents were 10 % aged under 35 years, 31 % aged 35–45 years, 44 % were aged 46–55 years and 16 % were over 55 years old. Fifty-four percent of the GPs were female (222/409) and a majority (62 %) of responders were qualified for 20 or more years. The age and sex of the responders was broadly comparable with the demographic profile of all GPs in the West Midlands.

### Use of prescribing formularies

Eighty-six percent (314/366) of respondents reported that they use antibiotic prescribing formularies to guide prescribing decisions. The majority of these respondents (73 %; 269/366) stated that they used a formulary provided by their PCT; with 45 (12 %) reporting using more than one formulary (Table 2).

**Table 2:** Reported source of antibiotic prescribing formularies/prescribing guidance used by survey respondents (*N* = 352)

Source of antibiotic formulary	Number using source^b^
Primary Care Trust^a^	269
British National Formulary	46
Local area prescribing committee	17
Practice formulary	13
Local NHS Microbiology department	6
NHS Hospital/Trust	4
Health Protection Agency (now part of Public Health England)	3
NICE	1

^a^On April 2013, PCTs were replaced by Clinical Commissioning Groups

^b^Note some respondents mentioned more than one source

Thirty-four percent (123/366) had reviewed compliance with existing policy for the management of UTI in the last 12 months prior to the survey.

### Influence of laboratory antimicrobial susceptibility results on antibiotic prescribing

Two hundred and fifty (70 %) respondents indicated that susceptibility results ‘always’ or ‘frequently’ influenced their antibiotic prescribing decisions for UTI. There was a significant difference (79 vs. 68 %; *p* = 0.021) between female and male GPs in the use of laboratory results to guide prescribing following treatment failure (Table 3). Only 6/362 (2 %) GPs reported that laboratory results infrequently or never influenced their prescribing in the case of reported resistance to initial therapy.

**Table 3:** Influence of laboratory results on antibiotic prescribing decision (number that would prescribe/number of respondents)

	Male				Female			
	Always	Frequently	Infrequently	Never	Always	Frequently	Infrequently	Never
General prescribing	22 % (37/167)	46 % (77/167)	25 % (41/167)	7 % (11/167)	21 % (40/190)	51 % (97/190)	22 % (42/190)	6 % (12/190)
In the case of a treatment failure	68 % (114/168)	29 % (49/168)	2 % (3/168)	1 % (1/168)	79 % (154/195)	19 % (38/195)	1 % (2/195)	1 % (1/195)
When resistance is reported to initial prescribed agent	81 % (136/168)	16 % (27/168)	2 % (4/168)	1 % (1/168)	86 % (168/195)	13 % (26/195)	1 % (1/195)	0 % (0/195)

### Factors influencing GPs decision to send urine specimens for diagnostic microbiology

Half (183/366) of the respondents reported that their practice had a policy to guide urine sampling for microbiological examination.

There was considerable variation among respondents regarding the approximate proportion of clinical consultations for suspected UTI that resulted in a urine specimen being sent for diagnostic microbiology (median 50 %, IQR 30 to 75 %).

### Clinical scenarios

In scenarios A, C and D (Table 4) the majority of GPs would submit a urine specimen for diagnostic microbiology (98, 98 and 97 % respectively), which is in-line with Public Health England (PHE) national guidance [16]. In scenario B, 40 % of GPs indicated that they would submit a urine specimen for microbiological testing, even though PHE guidance recommends samples should not be sent for examination routinely for uncomplicated UTI in female adults <65 years of age. There was a difference in urine sampling between genders for the catheterised asymptomatic elderly female scenario (scenario E) with 32 % of male GPs indicating they would submit a sample, compared to 43 % of female GPs (*p* = 0.034), whereas PHE guidance recommends that urine specimens should only be sent for examination in catheterized patients when features of systemic infection are observed.

**Table 4:** Count and percentage of GPs requesting urine samples and prescribing antibiotics for each clinical scenario

Clinical scenarios	Number (%) of GPs requesting a specimen	Number (%) of GPs that would prescribe an antibiotic
A. Treatment failure in a young women	344/352 (98 %)	284/353 (80 %)
B. Probable uncomplicated UTI	144/359 (40 %)	270/345 (78 %)
C. Probable UTI in an adult male	348/354 (98 %)	344/352 (98 %)
D. Possible asymptomatic UTI in pregnancy	341/353 (97 %)	129/352 (37 %)
E. Catheterised asymptomatic elderly female	134/354 (38 %)	5/348 (1 %)

The majority of GPs follow PHE guidance [16] by prescribing an antibiotic empirically for probable treatment failure (scenario A, 80 %), suspected uncomplicated UTI (scenario B, 78 %) and probable UTI in a male adult (scenario C, 98 %) (Table 4).

There was significant variation between male and female GPs for prescribing antibiotics in the suspected UTI in pregnancy scenario (scenario D) where 43 % of female GPs would prescribe compared to 30 % of male GPs (*p* = 0.0123).

One hundred and four (104/409, 25 %) GPs entered additional free text comments.

The main themes emerging from the analyses was the use of urinary dipstick test to investigate UTI in some of the scenarios presented, particularly scenario A (55/104, 53 %); the need to gather additional clinical information (15/104,14 %); inclination to send urine specimens by default (14/104, 13 %), and influence of the timing of the consultation in determining whether to take a specimen due to specimen transport issues (8/104, 8 %).

## Discussion

We consider this to be the first study examining the role of specific organisational and behavioral factors in the observed variation of urine sampling for diagnostic microbiology, and antibiotic prescribing for patients with UTIs among GPs serving a region of England. We found that the existence of a practice policy to guide sampling for diagnostic microbiology varied considerably across the region, as did the proportion of GPs that would submit a specimen and/or prescribe an antibiotic in a number of clinical scenarios. Understanding GPs knowledge and attitude towards the management of UTI within a region will aid interpretation of local AMR surveillance data, help guide appropriate use of regional laboratory resources and inform targeted interventions to promote antimicrobial stewardship.

A commonly cited issue in interpreting routinely reported AMR data from community settings is sampling bias, which may lead to observed levels of resistance that overestimate the burden of AMR in the general population. Only half of the GPs who responded reported having a practice policy to guide clinical sampling for diagnostic microbiology. There was considerable variation in the estimated proportion of clinical consultations for suspected UTI in which a urine specimen is sent for diagnostic microbiology. However we found that the response was broadly consistent for the scenarios involving: treatment failure, probable UTI in an adult male and possible UTI in pregnancy, and therefore using clinical scenarios may provide a more reliable insight into GP sampling practice than relying on a general view of GPs prescribing habits [4].

We found 40 % of GPs would submit a sample for diagnosis of the most commonly encountered presentation of uncomplicated UTIs, although PHE guidance recommends not sending urine samples for this presentation [16, 17]. This is a similar finding to a study in Wales in 2006 that found 56 % of randomly selected GPs would submit a urine specimen for probable uncomplicated UTI [4]. Also PHE guidance for management of UTIs in catheterised patients recommends that a urine sample should only be sent if there are signs of systemic infection [16], however 38 % of respondents would send a urine specimen in the catheterised asymptomatic elderly female scenario (Table 4). These results indicate non-compliance with guidance for certain clinical scenarios and a degree of inappropriate microbiological testing. A German study in 2005 concluded that most patients in their study were not treated according to current guidelines and for half the patients the decision to prescribe an antibiotic or the antibiotic prescribed was inappropriate [18].

It is plausible that this non-compliance with the guidance may be driven by ambiguity in the advice provided by existing national guidance. The National Institute for Health and Care Excellence (NICE) Clinical Knowledge Summaries advise that a urine sample should be sent to the laboratory for all women with suspected UTI associated with visible or non-visible haematuria [17]; however PHE guidance advises that urine samples should not be routinely submitted from women <65 of age, and if there are signs of UTI, including haematuria, then only empirical treatment should be given [16]. We recommend that these guidelines are reviewed so unambiguous evidence based guidance is made available to GPs.

Whilst acknowledging the importance of autonomy in clinical decision making, there is value in developing and utilising standardised, evidence based sampling policies to ensure that diagnostic and treatment decisions are both clinically effective and cost-effective [5]. Increasingly limited healthcare resources make a compelling case for standardising sampling policies, but this will only be achieved with consensus between microbiologists, community clinicians and policy makers.

The majority of respondents in our survey used local prescribing formularies produced by their PCTs. As PCTs were abolished at the end of March 2013 and replaced with Clinical Commissioning Groups (CCGs), we are unsure whether these formularies have been updated and are still being utilised. We recommend that CCGs work with their GP practices to review and update existing formularies.

A small proportion of respondents (14 %) indicated that they do not use a prescribing formulary to guide treatment decisions. We cannot tell from our survey if the non-utilisation of a formulary by these GPs results in inappropriate prescribing but we recommend that this issue be routinely assessed through the regular auditing and feedback of individual prescribing patterns and implementation of other interventions to address inappropriate prescribing as part of a wider antimicrobial stewardship programme.

The symptoms of UTI are often distressing to the patient, requiring immediate empirical therapy [19]. In our clinical scenarios most GPs would prescribe an antibiotic empirically for scenarios A, B and C (Table 4), which is in-line with national PHE guidance [16]; although it was surprising to find that a fifth of GP respondents would not prescribe an antibiotic in the treatment failure scenario (scenario A) given the presence of worsening symptoms.

Just over a third of our survey respondents indicated that they would prescribe an antibiotic for a suspected asymptomatic UTI in pregnancy (scenario D); even though national guidance recommends that treatment should only be considered after bacteruria is confirmed by culture [16].

We found the gender of the GP was a factor in the responses to some of the survey questions, with a greater proportion of female GPs reporting being influenced by laboratory results, taking specimens and prescribing in scenarios D and E.

A possible explanation may be differences in patient empathy with particular patients groups or difference in the desire to meet patient expectations [20]. A large English study in 2009 found a higher proportion of male GPs prescribing antibiotics in the community, and suggested male GPs perceive a greater pressure from patients to prescribe [8]. We therefore suggest that further behavioural studies are required to better understand variation in prescribing between genders and help inform the design of interventions aimed at changing prescribing habits.

There were some limitations to our study. The low response rate raises the possibility of non-response bias and its potential effect on the external validity of our study. We believe any effect on our estimates and the generalisability of our findings is low given that the demographic profile of our respondents is similar to that of all GPs in the West Midlands. In the free text comments, 3 GP respondents indicated that they may delay prescribing in some of the clinical scenarios; however the ‘yes’ or ‘no’ response options to these questions prevented the capture of this information.

Our analyses and interpretation of the free text comments may not be representative of the cohort of respondents as the number of comments was relatively small. However emerging themes from the analysis of these comments suggests that some GPs may be more inclined to send urine specimens by default. This needs to be explored further using alternative qualitative research methods such as focus groups.

For our next steps we intend to survey CCGs to determine whether antibiotic prescribing formularies developed by the PCTs are still being used and updated since the abolition of PCTs. We are also currently exploring the use of mobile device technologies to deliver timely localised AMR surveillance data and national prescribing guidance directly to clinicians in community settings and healthcare commissioners to support the management of UTI.

## Conclusion

The delivery of clinical care of consistent high quality will benefit from the implementation of antimicrobial stewardship programmes in community settings that include prescribing formularies based on local AMR surveillance and unambiguous national guidance on the management of infections. These, and policies to guide clinical specimen sampling will also facilitate the cost-effective use of available laboratory, and other finite healthcare resources.

## Additional file

Additional file 1: Survey questionnaire. (PDF 403 kb)

## Abbreviations

AMR: antimicrobial resistance
CCG: Clinical Commissioning Groups
GP: general practitioner
NICE: National Institute for Health and Care Excellence
PCT: Primary Care Trust
PHE: Public Health England
UTI: urinary tract infection
