# Supplementary material for: Use of antimicrobial resistance information and prescribing guidance for management of urinary tract infections: survey of general practitioners in the West Midlands
Source: BMC Infect Dis. 2016 May 24;16:226. doi: 10.1186/s12879-016-1559-2 (PMC4877747; doi:10.1186/s12879-016-1559-2)
Supplement: Additional file 1: — Survey questionnaire. (PDF 403 kb) [file 12879_2016_1559_MOESM1_ESM.pdf]

Management of community UTI

Survey logo

Welcome to this HPA Survey

The HPA Regional Epidemiology Unit is developing an Antimicrobial Resistance (AMR) surveillance bulletin for GP's in the West Midlands. To help understand potential variation in collection of samples for microbiological investigation and antimicrobial prescribing habits we would be very grateful if you could complete this short survey on the management of UTI.

1. Please provide the name of your practice (primary if you have more than one)*

2. Please enter your national Practice Code or if not known the Practice Postcode

	National Practice Code (e.g. M123456)	OR Practice Post Code
*		

3. Your age?*

○ <35 years ○ 35-45 years ○ 46-55 years ○ >55 years

4. Please can you enter the number of years since qualified.

5. Your gender?*

○ Male ○ Female

Management of community UTI

Survey logo

6. Is there a practice policy or protocol for sending urine specimens for microbiological examination?*

○ Yes ○ No

7. Does your practice use specific prescribing formularies ?*
If YES then please state the source of this guidance (e.g. HPA, PCT, BNF etc)

○ Yes ○ No

8. If the answer to question 7 is YES then please state the source of the prescribing formularie used in your practice (e.g. PCT, HPA, BNF)

9. Do laboratory antimicrobial susceptibility results for urinary isolates influence your antibiotic prescribing for:

	Always	Frequently	Infrequently	Never
General empirical prescribing:	○	○	○	○
In the case of a treatment failure:	○	○	○	○
When resistance is reported to initial prescribed agent:	○	○	○	○

10. Based on your experience of treating patients presenting with clinically suspected UTI what approximate proportion would you request a urine sample for microbiological examination [%]?* The value must be between 0 and 100, inclusive.

%

11. Has your practice reviewed the management of urinary tract infections within the last 12 months?*

○ Yes ○ No

Management of community UTI

Survey logo

For each of the scenario's below please state whether (a) a urine sample would be taken for microbiological examination and (b) an antibiotic agent prescribed (if prescribed empirically)

12. Case 1. A 20 year old lady re-attends surgery and complains that the loin pain and frequent urination symptoms reported to you the previous week had worsened despite finishing a complete course of trimethoprim (no sample was taken previously).

	Yes	No
Would you collect a urine sample for microbiological examination?	☐	☐
Would you prescribe an antibiotic?	☐	☐

13. Case 3. A 43 year old woman complains of pain passing urine and frequency. She feels well otherwise and has not previously been treated for a UTI.

	Yes	No
Would you collect a urine sample for microbiological examination?	☐	☐
Would you prescribe an antibiotic?	☐	☐

14. Case 4. A 51 year-old man attends your surgery complaining of pain passing urine and perineal tenderness. On examination you find suprapubic tenderness and a temperature of 38.5 C is measured.

	Yes	No
Would you collect a urine sample for microbiological examination?	☐	☐
Would you prescribe an antibiotic?	☐	☐

15. Case 5. During a routine antenatal clinic an 18 year old girl who is 20 weeks pregnant produces a cloudy urine sample. She reports no symptoms or discomfort. The urine dipstick tests positive for nitrite but negative for leukocytes and protein.

	Yes	No
Would you collect a urine sample for microbiological examination?	☐	☐
Would you prescribe an antibiotic?	☐	☐

16. Case 6. You visit an 82 year old female in a nursing home. She is catheterised, afebrile and has no symptoms but the staff inform you that the urine is cloudy.

	Yes	No
Would you collect a urine sample for microbiological examination?	☐	☐
Would you prescribe an antibiotic?	☐	☐

17. Please let us know if you have any additional comments regarding this survey.

Please enter any comments in the box below.
